# Toward an open access genomics database of South Africans: ethical considerations

**DOI:** 10.3389/fgene.2023.1166029

**Published:** 2023-05-16

**Authors:** Amy Gooden, Donrich Thaldar

**Affiliations:** ^1^ School of Law, University of KwaZulu-Natal, Durban, South Africa; ^2^ Petrie-Flom Center for Health Law Policy, Biotechnology and Bioethics, Harvard Law School, Cambridge, MA, United States

**Keywords:** autonomy, database, genomics research, open access, open consent, privacy, South Africa, Ubuntu

## Abstract

Genomics research holds the potential to improve healthcare. Yet, a very low percentage of the genomic data used in genomics research internationally relates to persons of African origin. Establishing a large-scale, open access genomics database of South Africans may contribute to solving this problem. However, this raises various ethics concerns, including privacy expectations and informed consent. The concept of *open consent* offers a potential solution to these concerns by (a) being explicit about the research participant’s data being in the public domain and the associated privacy risks, and (b) setting a higher-than-usual benchmark for informed consent by making use of the objective assessment of prospective research participants’ understanding. Furthermore, in the South African context—where local culture is infused with Ubuntu and its relational view of personhood—community engagement is vital for establishing and maintaining an open access genomics database of South Africans. The South African National Health Research Ethics Council is called upon to provide guidelines for genomics researchers—based on open consent and community engagement—on how to plan and implement open access genomics projects.

## 1 Introduction

Genomics research holds much potential to improve healthcare. Humanity’s understanding of the human genome and its role in health and disease has grown, with decreasing costs and technological advances leading to the proliferation of genome sequencing (Mattick et al., 2014), and with it, genomics research. In recent years, genomics research has burgeoned, with many institutions—both local and international—undertaking such research. However, this genomics research is not without its bioethical concerns. Foremost among these are research participant privacy protection and equitable data sharing among researchers. Moreover, from a developing global perspective, there is also the concern that only a very low percentage of the genomic data used in genomics research internationally relates to persons of African origin (Bentley et al., 2017; Bentley et al., 2020; Jackson, 2020; Adepoju, 2022). While some Northern Hemisphere countries have initiated the establishment of large-scale genome–phenome databases of their populations—most prominently UK Biobank (genetic data of 500,000 individuals and health information) (UK Biobank, 2022), BioBank Japan (260,000 individuals genomes and clinical information) (BioBank Japan, 2021), the China Kadoorie Biobank (512,000 individuals and health survey data) (China Kadoorie Biobank, n.d.), and the Estonian Biobank (200,000 individuals’ genomes as well as genetic and phenotype data) (Estonian Biobank, 2020)—the Global South appears to lag behind. The Southern African Human Genome Programme, launched in 2011 (Pepper, 2011), only sequenced the genomes of 24 individuals and lacks health data. In respect of accessibility, these databases generally require an application to be made and permission granted, before data can be accessed. For example, access to the Southern African Human Genome Project database is subjected to Data Access Committee (DAC) approval. If one’s purpose is to radically increase the data sharing of research participants of African origin in global genomics research projects, how should a large-scale South African genome–phenome database be structured?

We propose an *open access* database—freely available to genomics researchers all over the world—comprising individual-level genome–phenome data of tens (or hundreds) of thousands of South Africans. Thaldar et al. showed that there is indeed a *legal* pathway to make such an open access database a reality Thaldar et al., (forthcoming). In this article, we plot the landmarks for an *ethics* pathway. We start by sketching the broader policy and governance context, before analyzing the ethics issues at the core of an open access genomics project.

## 2 Background

### 2.1 Genomics research: progress *versus* privacy

Genomics research requires a balance to be struck between sharing data to advance scientific progress and safeguarding individual privacy (National Human Genome Research Institute, 2020). The unique nature of genomic information and increased data sharing have heightened privacy risks—for research participants as well as those genetically related to them (Kaye, 2012). This is because there are serious risks associated with both the publication of identifiable genomic data and the sharing of supposedly “de-identified” genomic data, which may violate the privacy of individuals and their relatives (Bonomi et al., 2020).

The term *de-identification* in South African law, in terms of section 1 of the Protection of Personal Information Act 4 of 2013 (POPIA), is defined as the deletion of any information that (a) identifies the data subject, (b) can be used or manipulated by a reasonably foreseeable method to identify the data subject, or (c) can be linked by a reasonably foreseeable method to other information that identifies the data subject. Given that a genomic sequence can always be compared to other genomic sequences to find a match that can potentially identify the data subject, it is unlikely that such genomic data can ever be *de-identified*, as contemplated in South African law (Townsend and Thaldar, 2019; Thaldar and Townsend, 2021; Thaldar et al., 2022). In fact, there are many examples of instances where supposedly “de-identified” genomic data have been re-identified using different techniques and processes (Altman, 2009; Resnik, 2009; Malin et al., 2010; Kaye, 2012; Gymrek et al., 2013; Erlich et al., 2014; Pereira et al., 2014; Pike, 2015; Shapiro, 2015; Hansson et al., 2016; Cacchio, 2018). The concern of re-identification includes risks such as genetic discrimination, especially in the insurance and employment contexts (Lynch et al., 2011); discrimination on the grounds of race, sex, and ethnicity (Erlich et al., 2014; ASSAf and DST, 2018); revealing of certain genetic predispositions, as well as other delicate health and personal information (Shapiro, 2015); the discovery of family relationships (Erlich et al., 2014); and the impact that it may have on social standing and self-image (Lunshof et al., 2008). Ultimately, this may hamper genomics research by deterring data collection and sharing (Bonomi et al., 2020).

Taking this into account, some solutions have been proposed. Organizational measures include access control systems, passwords, access agreements, and material (or data) transfer agreements. Technical measures encompass encryption (Rafter, 2022), firewalls (Ioannidis et al., 2000), virtual private networks (VPNs), and antimalware (Rosencrance, n.d.; Staunton et al., 2021). Yet, each of these methods has their own issues. For example, encryption—which is a popular method used to securely transfer and store sensitive information, like genomic data—requires infrastructure, time, and computational resources, depending on the size of the data (Carter, 2019). Additionally, because encrypted data need to be decrypted in order for researchers to be able to work with it, and because that data (in its decrypted state) are vulnerable to theft and disclosure, the type of encryption commonly used fails to fully safeguard data (Gil, 2020).

### 2.2 Amplifying the progress–privacy dichotomy in genomics research: open science

Genomics research must also be seen within the broader context of the open science movement. Although open science has been variously defined, it commonly denotes the “(a) full, frank, and timely publication of results, (b) absence of intellectual property restrictions, and (c) radically increased pre- and post-publication transparency of data, activities, and deliberations within research groups” (Maurer, 2003; Caso and Ducato, 2014). Open science is perceived to hold great benefits in that it promotes efficiency by reducing the duplication and cost of work, as well as in gathering, producing, transferring, and re-using data (OECD, n.d.); enhances quality by allowing broader and thus greater accuracy in the verification of results, as well as making research more reproducible (OECD, n.d.); promotes disclosure, engagement, and trust in science (Bueno de la Fuente, n.d.); fosters innovation through increased access to research (Allen and Mehler, 2019); assists in both domestic and international collaboration (Allen and Mehler, 2019); and accelerates research and discoveries that benefit society (OECD, n.d.).

The open science movement was exemplified by the Human Genome Project (HGP), which consisted of both international and cross-disciplinary collaboration, and succeeded 2 decades ago in mapping and sequencing the human genome. It promoted the open sharing of DNA sequencing information and the open access of software used in analyses. The HGP has had significant long-term effects on the fields of biology and medicine, specifically in terms of the development of proteomics (Ramachandran et al., 2021), furthering the understanding of evolution, and the discovery and classification of a “parts list” of nearly all human genes (Ramachandran et al., 2021). Since then, various other genomics research projects have adopted an open science approach. These include the International HapMap Project (HapMap) (National Human Genome Research Institute, 2012; Ramachandran et al., 2021) and the Encyclopedia of DNA Elements (ENCODE) Project (National Human Genome Research Institute, 2021; Ramachandran et al., 2021). This shows the value of collaboration and open access and sharing of data in advancing genomics research, relevant discoveries, and ultimately bettering population health.

However, open science’s commitment to open access and sharing of data has clear privacy implications. While the open science movement promises to optimize the benefits of genomics research, it also amplifies the potential risks to the privacy of research participants. Can these apparently opposing interests be reconciled? In the next section, we investigate this question by focusing on the fulcrum of contemporary human participant research: informed consent.

## 3 Analysis

### 3.1 Informed consent and its bedrock: autonomy

Research with human participants must adhere to various legal and ethical standards in order to protect individuals and ensure compliance with specified guidelines (Manandhar and Joshi, 2020). An important aspect is *consent*. When consent is granted, it assumes that the individual is aware of, and understands, what he or she is consenting to—in other words, it assumes that the individual is *informed* (Townsend et al., 2019). In order for consent to be *informed*, individuals must be provided with adequate and understandable information, including the possible advantages and drawbacks (McGuire and Beskow, 2010; HPCSA, 2016; Manandhar and Joshi, 2020). Instead of being viewed as a mechanistic formality where documents are read and signed, informed consent should be dynamic, involving information exchange and decision-making between the researcher and participant (Manandhar and Joshi, 2020) that allows individuals to understand the nature of the research and its risks, management of their samples and data, and safeguarding of their information (Chow-White et al., 2015; Manandhar and Joshi, 2020). From an ethics perspective, informed consent serves the value of *autonomy* by requiring individuals to *voluntarily* agree to a process or procedure based on sufficient information and knowledge (Hamvas et al., 2004).

In South Africa, autonomy as a value is understood through the lens of local culture—in particular through the lens of Ubuntu. Ubuntu is the hallmark of the philosophical thinking of communities and persons in southern Africa and is central to the political culture of post-apartheid South Africa (Bennett et al., 2018). The Ubuntu ethic is premised on the idea that humans are inherently social creatures that live interrelated lives and humans require other humans in order to live a good life; hence, members of a human community owe reciprocal obligations to each other aimed at achieving harmonious relations in society (Munyaka and Mothlabi, 2009). This idea is often expressed by the Nguni expression “umuntu ngumuntu ngabantu,” which can be translated as “a person is a person through other persons” (Mokgoro, 1998; Shutte, 2001; Metz, 2011). Ubuntu is a relational ethic that values harmonious relationships and emphasizes community, caring, and unity (Behrens, 2013). Metz (2010) elaborates on Ubuntu as follows: “an action is right just insofar as it is a way of living harmoniously or prizing communal relationships, ones in which people identify with each other and exhibit solidarity with one another; otherwise, an action is wrong.” However, not only does Ubuntu acknowledge the relational nature of persons, it also involves respecting individuality as this allows relationships with individuals and communities to flourish (Prozesky, 2009; Shozi, 2021). This entails recognizing and seeking to balance the interests of the community and the individual, rather than viewing one as more important than the other. Accordingly, when research projects that involve a community in South Africa are planned, it is ethically—and legally—required that the researchers consult with the community prior to engaging with community members on an individual level.

But how does informed consent—and therefore autonomy, understood through the relational lens of Ubuntu—relate to privacy?

### 3.2 Autonomy and privacy

Privacy exists in various dimensions (Leino-Kilpi et al., 2001; Ayres and Ribeiro, 2018), including *informational* privacy, which encompasses the right of an individual to choose when, how, and to what extent their information is shared with others (Leino-Kilpi et al., 2001); *social* privacy, which entails an individual’s capability to guide social contacts, and includes freedom from exchanges with others as well as any coercion on their chosen path (Leino-Kilpi et al., 2001); *psychological* privacy, which concerns an individual’s ability to control cognitive functions, create values, and decide when and with whom they share thoughts or information (Leino-Kilpi et al., 2001); and *physical* privacy, which refers to how physically available an individual is to others (Leino-Kilpi et al., 2001).

Given the nature and use of genomic data, *informational* privacy is the relevant dimension in the current context. At the heart of the protection of informational privacy is *autonomous choice*: persons themselves ought to decide when, and under what conditions, information about themselves may be disclosed, as well as the ambit of disclosure. This means that while the safeguarding of research participants’ genomic data may be the ethical default position, research participants ought to be free to waive such protection and make their genomic data open access. Accordingly, as a matter of principle, the notion that research participants’ genomic data *must always* be safeguarded from being made public and identified is mistaken. Also, in practice, it cannot merely be assumed that research participants would always *want* to exercise their right to privacy in respect of their genomic data—this is not only overly paternalistic toward research participants but also fails to account for Ubuntu-inspired persons who are willing to forego individual privacy for the benefit of their community.

Still, if research participants are requested, as part of the informed consent process of a research project, to elect not to exercise their privacy rights and make their genomic data open access, they are at increased risk of the possible adverse consequences associated with identified genomic data mentioned previously. This cannot be taken lightly. What is the solution?

### 3.3 Open consent

This was also the question that confronted the Personal Genome Project (PGP) when it was launched in 2005 at Harvard University with the aim of generating publicly accessible genome, health, and trait data without active efforts to de-identify such data (Angrist, 2009). The PGP adopted what it perceived as an honest and transparent approach, where participants were “truly informed” regarding the nature of the research (Ball et al., 2014; Cheung, 2018), rather than merely being required to sign long and incomprehensible consent forms (Angrist, 2009). This led to the development of *open consent* (Lunshof et al., 2008). This involves individuals donating and sharing their data for research with no assurances regarding its anonymity, privacy, or confidentiality (Lunshof et al., 2008). Instead, open consent entails that participants agree to their genotype–phenotype data being openly accessible, while being made aware of the benefits and risks of participation (including that some risks may by unanticipated) (Lunshof et al., 2010), access to samples and data, erosion of privacy and confidentiality, and possible re-identification (Lunshof et al., 2010; Budin-Ljøsne et al., 2017).

Importantly, to ensure that consent is truly informed, the PGP (a) provides prospective participants with resource material on the project, the use of samples and data, and risks to privacy, and (b) requires prospective participants to undergo an online entrance assessment in which their understanding of this resource material is objectively assessed (Angrist, 2009). A prospective participant must obtain full marks for this assessment before being accepted as a participant in the PGP (Angrist, 2009; Zarate et al., 2016; Cheung, 2018; Dankar et al., 2019). By significantly raising the bar for *informed* consent, objective assessment compensates for the increased risk entailed by making genomic data open access.

In essence, therefore, open consent is a form of blanket consent to making one’s data open access, coupled with an objective assessment of whether the consent is truly informed. Accordingly, open consent can be perceived as a way to reconcile autonomy with open science—and the benefits associated with it.

### 3.4 Using objective assessment in consenting research participants

The use of objective assessments as a part of informed consent is not new or unique to the PGP. For example, Kadam (2017) observes that “participants may have diverse learning abilities and educational backgrounds,” making the evaluation of participant’s informed consent comprehension prior to them consenting fundamental (Kadam, 2017). In 1998, Jimison et al. (1998) considered the use of multimedia in order to obtain informed consent, part of which included self-test questions. “Teach back methods” (Kadam, 2017) and questionnaires (Buccini et al., 2009; Kadam, 2017) have also been utilized to assess participant understanding (Kadam, 2017). Methods to assess informed consent comprehension—such as (a) the Deaconess Informed Consent Comprehension Test (DICCT) (Miller et al., 1996; Buccini et al., 2009; Burgess et al., 2019), (b) the Quality of Informed Consent (QuIC) questionnaire (Joffe et al., 2001; Buccini et al., 2009; Burgess et al., 2019), and (c) the Brief Informed Consent Protocol (BICEP) (Sugarman et al., 2005; Buccini et al., 2009; Burgess et al., 2019)—have also been developed (Buccini et al., 2009; Kadam, 2017). However, these methods have various limitations. For example, the QuIC questionnaire was developed for cancer clinical trials and may therefore be ill-suited to other trials. The QuIC questionnaire and the DICCT are grounded on requirements for consent in the United States of America and may need to be modified for use in other countries (Buccini et al., 2009). Lastly, all three methods offer limited guidance regarding how to interpret and use prospective participants’ test results for the purpose of recruitment (Buccini et al., 2009).

In all of the aforementioned examples, enrolment is not necessarily contingent upon participants passing the assessment. Rather, it is merely used to determine whether participants are able to sufficiently understand the content of the project and what they are consenting to (Jimison et al., 1998). According to Burgess et al. (2019), although researchers recognize the relevance of testing participants’ informed consent comprehension, it is scarcely applied in practice—possibly due to ambiguity regarding the appropriate assessment mechanism, as well as how to manage participants who fail. The following questions arise: should such participants be prevented from enrolling in a research project? and Should researchers continue to explain and assess relevant information until all participants pass (Burgess et al., 2019)?

The PGP answers these questions by allowing prospective participants to take the online entrance assessment as many times as they like (Ball et al., 2014). It should be noted that prospective participants are not provided with information on which questions they had incorrect, but only their overall score. This, of course, increases the difficulty. In terms of the open consent model developed by the PGP, prospective participants are provided with study material prior to taking the enrolment assessment. Although no such material currently exists in South Africa, the material used by the PGP serves as a good basis that can be built upon and adapted to suit the South African research participant community.

### 3.5 Bringing open consent home to South Africa

Given that South African culture is permeated by Ubuntu and its relational view of personhood, it is important for researchers to engage with their research participant communities. Members of a community can offer unique insights which assist in ensuring that regard is had to the community prior to, and during, a research project (Musesengwa and Chimbari, 2017). In the research context, community engagement refers to various activities, including the distribution of information, consultation, empowerment, participatory decision-making, the formation of partnerships with stakeholders, and the provision of guidance by community leaders (Musesengwa and Chimbari, 2017; Akondeng et al., 2022). Community engagement is recognized as a crucial factor in the success of a research project (Akondeng et al., 2022; Black and Sykes, 2022). Community engagement promotes community participation and ensures an acknowledgment of community preferences by researchers (Akondeng et al., 2022).

Community engagement is not only beneficial for members of the community in which it enhances community understanding of the research, aids the informed consent process, and lessens possible exploitation (Ahmed and Palermo, 2010; Tindana et al., 2015; Musesengwa and Chimbari, 2017), but it can also assist researchers in understanding community preferences and developing communication and research approaches that are tailored to the community (Ahmed and Palermo, 2010; Black and Sykes, 2022). Community engagement should be continuous throughout, and following, the research (Tindana et al., 2015). Therefore, community engagement strategies should be included in the research design, application, monitoring, and feasibility (Akondeng et al., 2022). There is no one strategy that can be used when engaging with a community, and the most suitable approach depends on the purpose of the research and the community involved (H3Africa Community Engagement Working Group for the Human Heredity and Health H3Africa Consortium, 2017; Tindana et al., 2015; Dickert and Sugarman, 2005). However, community engagement strategies should be flexible and adaptable to both community and researcher needs (Musesengwa and Chimbari, 2017). Furthermore, because of the heterogeneity amongst communities, the results of community engagement may differ across contexts (Akondeng et al., 2022).

Three important aspects in community engagement are (one) information sharing, (two) community consultation, and (three) community involvement and collaboration (Tindana et al., 2015). In terms of (one), the sharing of information is central to all research but becomes especially important in the context of genomics research, where terminology and processes are often complex. Thus, providing information is central to building rapport and community empowerment. Certain research projects may require educational materials to explain vital parts of a research project (Tindana et al., 2015). In terms of (two), community consultation entails gathering feedback from the community regarding aspects of the research in order to consider the community’s interests. Consultation may occur with different groups within the community, including community leaders and representatives, community advisory boards, and research ethics committees, before informed consent is sought from individuals (Tindana et al., 2015; Musesengwa and Chimbari, 2017). In terms of (three), community involvement encourages the formation of genuine and mutually respectful relationships between the researchers and the community (Tindana et al., 2015).

There are a range of strategies used to engage communities in research studies (Akondeng et al., 2022). For example, visual forms of communication and participation can strengthen communication (especially where language and literacy barriers are present), learning, and harmonize power and knowledge dynamics (Black and Sykes, 2022). Visual materials promote engagement through the discussion of opinions, ideas, and needs. This, in turn, can foster effective community engagement and involvement (Black and Sykes, 2022). Community meetings and focus group discussions can also be used to directly engage with the prospective participants and their communities (Tindana et al., 2015; Puerta et al., 2020). These can either consist of larger community gatherings (such as town hall meetings) or small groups within the community such as women’s groups or patient associations. These meetings aim to discuss the research and obtain individuals’ feedback. The outcomes of these meetings can serve to guide the research—for example, in terms of altering informed consent forms or the enrolment process (H3Africa Community Engagement Working Group for the Human Heredity and Health H3Africa Consortium, 2017) to make it more culturally appropriate. Going out into communities and interacting with them promotes and strengthens relationships between researchers and communities, and this goes a long way in ensuring that the researchers take the interests of the community into consideration and act in a manner that promotes Ubuntu.

## 4 Conclusion

An open access genomics database of South Africans, provided that it can successfully recruit on a large scale, will hold immense promise for health benefits for future generations of South Africans, such as precision medicine. Moreover, concerning global health research equity, it would make the genomic data of Africans freely available, hence offering a solution to increasing the shockingly low percentage of genomic data of persons of African origin used in genomics research globally. Open consent, adapted for a South African context, provides an ethics pathway for establishing such an open access genomics database in South Africa.

The South African National Health Research Ethics Council (NHREC), the statutory body responsible for setting national standards and norms for health research, is currently revising its guidelines. We call on the NHREC to give specific attention to open access genomics projects and provide clear and detailed guidelines for genomics researchers—based on open consent and community engagement—on how to plan and implement open access genomics projects.

## Data Availability

The original contributions presented in the study are included in the article/Supplementary Material. Further inquiries can be directed to the corresponding author.
